# Retraction: Inhibition of microRNA-218 reduces HIF-1α by targeting on Robo1 in mice aortic endothelial cells under intermittent hypoxia

**DOI:** 10.18632/oncotarget.28449

**Published:** 2023-05-26

**Authors:** Kai-Xiong Liu, Qin Chen, Gong-Ping Chen, Jian-Chai Huang, Jie-Feng Huang, Xin-Ru He, Ting Lin, Qi-Chang Lin

**Affiliations:** ^1^Department of Respiratory Disease, The First Affiliated Hospital, Fujian Medical University, China; ^2^Laboratory of Respiratory Disease of Fujian Medical University, Fuzhou, China; ^3^Fujian Provincial Sleep-disordered Breathing Clinic Center, Fuzhou, China; ^4^Integrated Chinese and Western Medicine Colleges, Fujian University of Traditional Chinese Medicine, Fuzhou, China

**This article has been retracted:** Images in Figure 4A are duplicates of those that appear in Figure 3B-1 and 5B-2 of an earlier published paper [1]. In addition, there are multiple internal image duplications. The authors were also unable to replicate certain experimental results. In light of this, all authors have agreed to the retraction of this paper from Oncotarget.

Original article: Oncotarget. 2017; 8:104359–104366. 104359-104366. https://doi.org/10.18632/oncotarget.22239
